# Metabolic Perturbations and Severe COVID-19 Disease: Implication of Molecular Pathways

**DOI:** 10.1155/2020/8896536

**Published:** 2020-11-28

**Authors:** Ersilia Nigro, Fabio Perrotta, Rita Polito, Vito D'Agnano, Filippo Scialò, Andrea Bianco, Aurora Daniele

**Affiliations:** ^1^Dipartimento di Scienze e Tecnologie Ambientali Biologiche Farmaceutiche, Università Degli Studi Della Campania “Luigi Vanvitelli”, Via G. Vivaldi 42, Caserta 81100, Italy; ^2^CEINGE-Biotecnologie Avanzate Scarl, Via G. Salvatore 486, Napoli 80145, Italy; ^3^Dipartimento di Medicina e Scienze Della Salute “V. Tiberio”, Università Del Molise, Campobasso 86100, Italy; ^4^Dipartimento di Scienze Mediche Traslazionali e Chirurgiche, Università Della Campania “L. Vanvitelli”, Napoli 80131, Italy

## Abstract

Coronavirus disease (COVID-19) is caused by SARS-CoV-2 virus, which can result in serious respiratory illnesses such as pneumonia leading to respiratory failure. It was first reported in Wuhan, Hubei, China, in December 2019 and rapidly spread globally, becoming a pandemic in March 2020. Among comorbidities observed in SARS-CoV-2 positive patients, hypertension (68.3%) and type 2-diabetes (30.1%) are the most frequent conditions. Although symptoms are highly heterogeneous (ranging from absence of symptoms to severe acute respiratory failure), patients with metabolic-associated diseases often experience worse COVID-19 outcomes. This review investigates the association between metabolic disorders and COVID-19 severity, exploring the molecular mechanisms potentially underlying this relationship and those that are responsible for more severe COVID-19 outcomes. In addition, the role of the main biological processes that may connect metabolic alterations to SARS-CoV-2 infection such as hyperglycemia, immune system deregulation, ACE-2 receptor modulation, and inflammatory response is described. The impact of metabolic disorders on the prognosis of COVID-19 has major implications in public health especially for countries affected by a high incidence of metabolic diseases.

## 1. Introduction

Cluster cases of pneumonia of unknown origin were described in December 2019 in Wuhan, Hubei Province, China; the cause was later identified as the novel coronavirus SARS-CoV-2 [1]. Subsequently, the spread of the novel SARS-CoV-2 coronavirus has reached pandemic levels as declared by the World Health Organization (WHO) and still represents a global threat for increased morbidity and mortality. WHO data about the pandemic spread on 2^nd^ November 2020 reported 46,166,182 confirmed cases worldwide since the start of the outbreak, and 1,196,326 deaths in 219 countries [2]. In Italy, 632.092 confirmed cases and 38.190 (6%) deaths have been registered according to the Istituto Superiore di Sanità up to November 2^nd^ [3].

Coronaviruses (CoVs) are a large family of enveloped viruses containing single-stranded positive-sense RNA. Previously, two different CoVs, severe acute respiratory syndrome CoV (SARS) and Middle East respiratory syndrome (MERS), outbreaks caused significant mortality in 2003 and 2009 [4]. SARS-CoV-2 virus entry is mediated by the viral spike protein that forms homotrimers and binds to human cells [5]. The protein spike (S) is composed of two subunits, S1 responsible for the binding to the host cell membrane (via receptor) and S2 that determines the fusion of the viral and cellular host membranes [5, 6]. Successively, host proteases cleave the S protein, irreversibly activating the S protein and the consequent entry of the virus [7]. The symptoms, which usually emerge between 2 and 14 days after infection, are strongly heterogeneous ranging from absence or mild symptoms to severe disease and death [1, 4]. The mechanisms and factors influencing the phenotypic manifestations of SARS-CoV-2 infection remain unclear. General health condition, age, and concomitant morbidities appear to determine clinical manifestation and adverse outcomes for SARS-CoV-2-induced pneumonia [6]. Severe and fatal SARS-CoV-2 infection occurs mainly in the elderly or patients with underlying clinical conditions. Indeed, most hospitalized patients, admitted to intensive care units for severe acute respiratory distress syndrome, exhibit comorbid conditions [8, 9]. Previous SARS-CoV and MERS-CoV epidemics also displayed worse outcomes and challenging management when comorbidities were present [10–12]. Emerging data document that SARS-CoV-2 infection is mainly associated with hypertension and/or metabolic disorders (the most common), cerebrovascular diseases, hepatitis B infections, chronic obstructive pulmonary disease, chronic kidney diseases, and malignancy [13, 14]. Individuals affected by type 2 diabetes and severe obesity (BMI ≥ 40 kg/m2) are more likely to be infected and are at high risk for complications and death from SARS-CoV-2 infection [15–19].

This review analyzes correlations between metabolic disorders, mainly obesity and type 2 diabetes, and SARS-CoV-2 infection looking at the underlying molecular mechanisms involved and their impact on prognosis.

## 2. Comorbidities in SARS-CoV-2 Infection

Initial data on SARS-CoV-2 infection documented that patients with preexisting metabolic and cardiovascular diseases experienced unfavorable outcomes [8, 20]. The influence of comorbidities in coronaviruses infections has been reported during previous SARS and MERS outbreaks [10, 21–28] (see Table 1). Chronic cardiovascular disorders and diabetes mellitus have been reported as the major comorbidities influencing prognosis during previous coronavirus epidemics [29]. A nationwide analysis on SARS-CoV-2 in China reported a 25.1% prevalence of at least 1 comorbidity among 1.590 patients tested positive for SARS-CoV-2. The authors found a proportion of 8.2% of diabetes, 16.9% of hypertension, and 57.3% of other cardiovascular diseases [13]. Likewise, comorbidities are highly prevalent among SARS-CoV-2 patients who experience a more severe clinical disease course. In Italy, according to the report of Istituto Superiore Sanità (ISS) based on available data on July 9^th^, 2020, among patients with SARS-CoV-2, the mean number of underlying diseases was 3.3 among 3857 patients dying in hospital. A single comorbidity was found in 13.2% patients, 2 comorbidities in 19.3%, 3 or more in 64% patients. Among common comorbidities observed in SARS-CoV-2 positive deceased patients, hypertension (66.2%), type 2-diabetes (29.8%), and ischemic heart disease (27.7%) are the most frequent conditions [1, 30, 31]. In addition, the overall proportion of hypertension and diabetes seems to be about twofold higher in ICU/severe cases than in non-ICU/severe counterparts [31] and although diabetes may not increase the risk of infection, the coexistence of this comorbidity among SARS-CoV-2 patients results in excess mortality [32].

The complex mechanisms linking hypertension and diabetes interact at different levels resulting in bidirectional interplay. Firstly, there are several shared biological mechanisms that influence this multifaceted network; they include unbalanced renin angiotensin aldosterone system homeostasis (RAAS), systemic proinflammatory status, elevated oxidative stress, and increased sympathetic nervous system (SNS) activation [32]. Secondly, perturbation of insulin sensitivity leads to an imbalance in insulin-mediated vasodilator/vasoconstrictor activities. This is associated with vascular stiffness, hypertrophy, fibrosis, and remodeling mainly driven through MAPK dependent signaling pathways. Indeed, insulin stimulates secretion and expression of vasoconstrictor mediators, including endothelin-1, PAI-1, and vascular cell adhesion molecule-1 resulting in increased vasoconstrictor effects [33–35]. Furthermore, obesity and the release of adipose released hormones influence the risk and severity of both hypertension and diabetes [36]. Likewise, both hypertensive status and disproportionate adiposity may alter the interaction between the pulmonary microenvironment, viral pathogenesis, and immune cell trafficking [32, 37].

Emerging data in SARS-CoV-2 indicate the importance of obesity in influencing clinical outcomes. Lighter and coworkers highlighted the impact of obesity in SARS-CoV-2 patients aged <60 years. Based on 3.615 subjects tested positive, the authors concluded that patients with a BMI between 30 and 34 were 2.0 (95% 1.6–2.6, *p* < 0.0001) and 1.8 (95% CI 1.2–2.7, *p*=0.006) times more likely to be admitted to an acute department and ICU, respectively, compared to individuals with a BMI < 30 [28, 35]. However, the comprehensive mechanisms interacting between the virus-induced local inflammation and secondary reactive damage induced from excessive cytokine storm in patients with comorbidities have not been fully clarified.

## 3. Molecular Mechanisms Linking Metabolic Diseases and SARS-CoV-2 Infection

It is plausible that more than one mechanism concurs in determining the relationship between metabolic diseases and SARS-CoV-2 resulting in elevated susceptibility to infection, as well as a worse prognosis of the disease. The interrelations between organs and mechanisms involved are complex; the main factors implicated seem to be as follows: hyperglycemia and immune system deregulation, ACE-2 receptor modulation, and inflammatory response.

### 3.1. Hyperglycemia, Adipose Tissue, and Immune Response Deregulation

Hyperglycemia has been associated not only with disease severity but also with death in patients with severe SARS-CoV-2 infection [36]. Interestingly, these findings are consistent with studies of patients infected with highly pathogenic avian influenza, as well as SARS and MERS where the presence of uncontrolled hyperglycemia was associated with poor outcomes. Multiple molecular mechanisms linking hyperglycemia and SARS-CoV-2 infection have been proposed including the regulation of the receptor expression used by the virus for cell entry, an altered immune response as well as an inflammatory echo.

Indeed, high blood glucose may increase viral entry and replication in vivo, possibly through the modulation of the ACE2 receptor [37–39]. We will further debate this aspect later in the review. Elevated glucose levels may also suppress the antiviral immune response increasing the severity of viral infections. Hyperglycemia affects the innate and adaptive immune responses at several levels: it reduces neutrophil degranulation, chemotaxis, and phagocytic activity; impairs complement activation; and inhibits lymphocyte proliferative response [40, 41]. As reported by Berbudi et al. hyperglycemia in diabetes is thought to cause dysfunction of the immune response which fails to control the spread of invading pathogens in diabetic subjects [42]. It has been reported that hyperglycemia strongly reduces the macrophagic activity of both neutrophils and macrophages exposing patients to infection [43]. During infection or inflammation, macrophages first exhibit the M1 phenotype to release TNF-*α*, IL-1*β*, IL-12, and IL-6 against the stimulus. However, if the M1 phase persists, it can result in direct tissue damage. Therefore, M2 macrophages secrete high amounts of IL-10 and TGF-*β* to suppress the inflammation, contribute to tissue repair, remodeling, vasculogenesis, and retain homeostasis [43]. In diabetes, M2 macrophages lose the capacity to suppress M1 macrophages activity promoting a chronic inflammation that makes diabetic subjects more susceptible to infections [43]. In addition, hyperglycemia activates Protein Kinase C inhibiting neutrophil migration, phagocytosis, superoxide production, and microbial killing. It can also induce Toll-like receptor expression inhibiting neutrophil functions and apoptosis [44]. As reported by Jafar et al. [44], high glucose concentrations decrease vascular dilation and increase permeability during the initial inflammatory responses, possibly through Protein Kinase C activation [44]. Furthermore, hyperglycemia can cause direct glycosylation of proteins and can alter the tertiary structure of complement; these changes inhibit immunoglobulin-mediated opsonization of bacteria and complement fixation to bacteria and decrease phagocytosis [44].

Previous evidence from other CoV models documented that MERS-CoV infection in a diabetic mouse model exhibited more severe disease and slower recovery due to delayed inflammation up to 21 days after infection [45]. Diabetic mice had fewer inflammatory monocyte/macrophages and CD4+ T cells which correlated with lower levels of TNF*α* and IL-6 but higher levels of IL-17a expression. In line with these results, patients with SARS-CoV-2 are characterized by low peripheral CD4+ and CD8+ T cells counts, with high levels of proinflammatory Th17 CD4 + T cells, as well as elevated cytokine levels [15, 46, 47]. Thus, these data suggest that the increased disease severity observed in individuals with SARS-CoV-2 infection and concomitant hyperglycemia is likely due to a dysregulated immune response, which results in more severe and prolonged pathology. Also, human studies have confirmed a strong correlation between hyperglycemia and worse outcome of SARS-CoV-2. Indeed, as reported by Bode et al. among 570 SARS-CoV-2 patients who died or were discharged, the mortality rate was 28.8% in 184 diabetes and/or uncontrolled hyperglycemia patients, compared with 6.2% of 386 patients without diabetes or hyperglycemia [48]. It is of interest that optimal glycemic control during hospitalization is associated with a reduction in risk of severe disease and death in patients with COVID-19, demonstrating that hyperglycemia remained a strong prognostic predictor of outcome in hospitalized patients with COVID-19. Furthermore, hyperglycemic COVID-19 patients versus normoglycemic ones displayed a higher cumulative incidence of severe disease [49]. Hyperglycemia may also affect pulmonary function, directly increasing glucose concentrations and vasculature permeability in airway secretions [36, 47] that facilitate the virus infection and replication [50]. A very recent work reports that COVID-19 patients with diabetes mellitus were more likely to have elevated inflammatory markers and hypercoagulability, accompanied by hypoproteinaemia and glucose and lipid metabolism disorders. As a consequent, COVID-19 combined with diabetes prolonged the time of detoxification in patients [51].

It is essential to establish a glycemic profile in SARS-CoV-2 patients to better control the clinical outcomes in patients with coexistent diabetes, obesity, and hyperglycemia. Obesity, as reported above, may represent another interesting factor associated with poor prognosis among COVID-19 patients. A complex cross talk between adipose tissue and the immune system may have a relevant implication for SARS-CoV-2 infection. In the physiological state, although the immune cells pervade adipose tissue, a balance between immune cells and adipocytes and, in turn, adipokines production is maintained [52]. In pathophysiological conditions, such as obesity, the adipose tissue is pervaded by an enormous number of immune cells leading to an imbalance in the production of adipokines such as adiponectin and leptin [53, 54]. In addition, these adipokines are involved in energy homeostasis, glucose balance, and insulin sensitivity enhancing insulin sensitivity and glucose uptake and increasing, in turn, GLUT-4 translocation [53, 55]. Understanding the mechanisms underlying interactions between adipocytes released hormones, immune deregulation, and comorbid related conditions may offer further insight into tracking the SARS-CoV-2 pathogenesis.

### 3.2. ACE2 Receptor: Physiological Mechanisms and Implication for SARS-CoV-2 Infection

Coronaviruses attachment and adhesion to human target cells are mediated by a spike (*S*) protein which protrudes from the viral surface [56, 57]. For SARS-CoV and SARS-CoV-2 a binding of the S1 region, named receptor-binding domain (RBD), through the angiotensin-converting enzyme 2 (ACE2) receptor on host cells has been shown. The monocarboxypeptidase ACE2 was originally identified in 2000 as a homolog of ACE receptor [58, 59] and, subsequently, the molecular structure has been extensively described [60]. ACE2 expression is broadly represented in different biological systems including airway and type II lung alveolar cells, oesophageal epithelial cells, enterocytes, cholangiocytes, myocardial cells, kidney proximal tubule cells, and bladder urothelial cells [61]. In human lungs, ACE2 generates angiotensin (I-VII) from angiotensin II by the cleavage of a single amino acid [62]. Ang-(I–VII) through Mas receptor (Mas1) activation is expressed on endothelial cells and results in vasodilatory, anti-inflammatory, and antifibrotic effects [63]. Interestingly, the interaction between SARS-CoV-2 and ACE2 induces the downregulation of ACE2 expression resulting in AngII accumulation with proinflammatory and profibrotic effects [64, 65]. In a small cohort study, levels of Ang II were found markedly increased in SARS-CoV-2 plasma samples [66]. Elevated Ang II levels were found in mice infected with SARS [65] or H7N9 virus resulting in worse clinical outcomes [57, 67, 68] Accumulation of AngII promotes cell apoptosis through the interaction with receptor AT1R [68]. Different signaling pathways may activate apoptotic stimuli. Firstly, exalted oxidative stress related to overactivation of the AngII/AT1R/NAPDHox axis has been correlated with cardiovascular disorders, mainly hypertension [69] and atherosclerosis [70]. Therefore, downstream generation of reactive oxygen species (ROS) promotes apoptosis through the release of CytC from damaged mitochondria [71], activation of caspase 3 [72], or p38MAPK/JNK cascade [73]. Furthermore, NF-kb activation and the transcription of cytokines such as interleukin-6, IL-1*β*, and tumor necrosis factor alpha (TNF *α*) have been also associated with proapoptotic signals [74]. High levels of AngII and proinflammatory cytokines can then act synergistically and increase the level of cyclooxigease 2 (COX2) with the consequent buildup of ROS and inflammatory prostaglandin E2 [75]. In hypertensive rats, low levels of ACE2 mRNA leading to reduced Ang-(I–VII)/Mas-1 signals have been documented as compared to nonhypertensive counterparts [76, 77]. Interestingly, polymorphisms for ACE2 were independently associated with increased hypertension susceptibility and cardiovascular complications in diabetes [76, 77]. The overactivation of these pathways can result in a state of hyperinflammation that is seen in the late phase of SARS-CoV-2 infected patients.

### 3.3. ACE2 Expression Modulation: Implication for Antihypertensive and Antidiabetic Treatments

ACE2 expression is notably modified by agents acting on RAS. RAS inhibitors are the cornerstone of therapies for many cardiovascular and renal diseases. These drugs are widely used also in diabetic patients for preventing cardiovascular remodeling and diabetic nephropathy. Recently, in a multicenter retrospective study including hospitalized COVID-19 patients with previously diagnosed hypertension, the use of ACE inhibitors (ACE-Is) and Angiotensin Receptor Blockers (ARBs) was associated with lower all-cause mortality (adjusted HR, 0.42; 95% CI, 0.19–0.92; *p*=0.03) and septic shock (adjusted HR, 0.36; 95% CI, 0.16–0.84; *p*=0.01). Similarly, in another study of COVID-19 patients with hypertension, poor outcomes were witnessed when compared to nonhypertensive patients; interestingly, patients under ARBs/ACE-Is were less likely to be admitted to ICU (9.3% vs. 22.9; *p*=0.061), with a lower death rate (4.7% vs. 13.3%; *p*=0.283) than treated with non-ACE-Is/ARBs agents, although these differences did not reach statistical significance [78].

Experimental and clinical models showed different responses to the administration of agents interfering with this regulatory axis: in particular ARBs and mineralocorticoid-receptor blockers seem to increase the levels of ACE2 expression [78, 79], while administration of ACE-Is, thus increasing cardiac ACE2 mRNA levels, did not result in enhanced ACE2 activity [79]. Early evidence of increased cardiac ACE2 expression was shown in the rat model. After myocardial infarction, the administration of ARBs (olmesartan or losartan) increased the ACE2 expression about threefold [80]. In another experimental model, losartan administration compensated ACE2 smoke-induced reduction therefore restoring the ACE/ACE2 ratio in the lung [81]. In another study, increased urinary ACE2 levels were observed in hypertensive patients treated with Olmesartan [82].

However, based on the above-discussed mechanisms linking ACE2 expression with local anti-inflammatory, antiproliferative, and antifibrotic proprieties, several international societies and associations have recommended against ACE-Is or sartans discontinuation in patients under chronic therapy [83].

Other antidiabetic agents potentially interfere with the regulation of the RAS. Thiazolidinediones are an important class of insulin sensitizers used in the treatment of type 2 diabetes. In diabetic patients, the molecular mechanisms of biological responses of thiazolidinediones are mediated through the modulation of Peroxisome Proliferators Activated Receptors (PPARs). Similar to ACEIs and ARBs, thiazolidinediones can upregulate ACE2 expression [84, 85] potentially exposing alveolar cells to SARS-CoV-2 infection. However, PPARs are mediators of inflammation with potential immunoregulatory characteristics. Their activation prompts a reduction in inflammatory cytokines that are highly involved in SARS-CoV-2 (i.e., IL6 and INF *γ*) [86]. Two different PPAR*γ* agonists, rosiglitazone and pioglitazone, are insulin-mimetic drugs used in type 2 diabetes with high overall potential in reducing influenza virus infection [86]. In mice infected with the influenza virus, pioglitazone administration resulted in improved survival based on anti-inflammatory properties [86, 87].

### 3.4. Increased Inflammation in SARS-CoV-2 Patients with Metabolic Disorders

It is known that subjects affected by metabolic disorders, such as obesity and diabetes, have increased risk of bacterial, mycotic, parasitic, and viral infections [88]. Drucker et al. documented that acute viral respiratory infection has been linked to the rapid development of transient insulin resistance, in both otherwise healthy euglycemic normal weight or overweight individuals [89, 90], therefore demonstrating that infectious diseases strongly increase mortality in diabetic subjects. Retrospective analysis showed that mortality was increased in older diabetic patients [91]. Diabetic disease is strongly associated with increased risk and worse outcomes for bacterial and viral infections attributed to a combination of dysregulated innate immunity and inflammatory responses [92, 93]. Furthermore, coronavirus infections may be complicated by secondary bacterial infections, reflecting the compromised epithelial barrier function in the lungs and the gastrointestinal tract in diabetic subjects [89].

During viral infection, the chronic inflammation and related cytokines production induce neutrophilia, coagulation activation, and kidney injury leading to the death of patients with SARS-CoV-2 [94, 95]. Several studies have shown that, in diabetic patients with SARS-CoV-2, absolute count of lymphocytes in peripheral blood is significantly lower, while the absolute count of neutrophils is remarkably higher [96]. In addition, diabetic subjects with SARS-CoV-2 have higher serum levels of various inflammatory-related biomarkers compared to nondiabetic patients with SARS-CoV-2 [57]. In particular, these subjects are characterized by elevated serum levels of IL-6, TNF*α*, serum ferritin, and C reactive protein (CRP). Among these, IL-6 is a predictor of disease severity and prognosis and its expression time is longer than other cytokines (TNF*α* and IL-1) [97]. Furthermore, Guo et al. reported that, in diabetic patients, there is also an increase in serum ferritin indicating the activation of the monocyte-macrophage system, which is a crucial part of the inflammatory storm [94, 97]. The authors concluded that patients with diabetes are susceptible to develop an inflammatory storm, which eventually leads to rapid deterioration of SARS-CoV-2 patients [97].

Finally, diabetes represents a noteworthy risk factor for impaired coagulative imbalance and platelet aggregation which might sustain the enhanced thromboembolic disorders observed in deceased patients with COVID-19 [98]. Several mechanisms linking inflammation and coagulative homeostasis in diabetic patients have been explored [95]. Firstly, D-dimer is increased by inflammation activating plasmin. Secondly, chronic inflammation and hypoxia activate thrombin and the activation of monocyte-macrophages causes secretion of a mass of tissue factors and activation of the exogenous coagulation pathway, which leads to an overall hypercoagulable state or even disseminated intravascular coagulation [94, 95]. In addition, elevated D-dimer levels are consistently reported and their gradual increase during disease course is particularly associated with disease worsening [99]. In conclusion, during the disease course, longitudinal evaluation of lymphocyte count dynamics and inflammatory indices, such as CRP, IL-6, and ferritin may help to identify cases with poor prognosis and prompt intervention in order to improve outcomes.

### 3.5. Cytokine Role in Metabolic-Mediated Effects in SARS-CoV-2

The uncontrolled production of IL-6 has been demonstrated to correlate with disease progression and severity [100], predicting respiratory failure in hospitalized symptomatic COVID-19 patients [101]. This could be accentuated in diabetic patients where low-grade inflammation, a characteristic of this pathology, can facilitate the cytokine storm caused by SARS-CoV-2 infection. Among the released cytokines, IL-6, IL-1B, and TNF*α* can have a major impact on glucose metabolism, insulin signaling, and induce cardiovascular complications [102]. For instance, CRP can be stimulated by the release of IL-6 [103]. CRP can in turn aggregate and induce the activation of complement and tissue factor initiating coagulation [104]. It is worth noting that diabetic patients are more prone to develop coagulopathy such as disseminated intravascular coagulation (DIC) [105], a pathology that could be exacerbated in combination with SARS-CoV-2 infection and result in severe outcomes [106]. A further IL-6 increase in COVID-19 diabetic patients can exacerbate insulin resistance by inhibiting the autophosphorylation of the insulin receptor and the activation of PI3K and AKT pathway [107, 108]. Like IL-6, increased levels of TNF*α* have also been shown to induce insulin resistance by impairing insulin signaling and glucose uptake in vivo [109]. TNF*α* can induce the translocation of NF-kb into the nucleus and promote the transcription and release of more cytokines generating a vicious cycle. Finally, the overproduction of IL-1B can lead to B-cell dysfunction and death through the induction of nitric oxidase synthase and the excessive production of nitric oxide [110]. Leptin, a proinflammatory cytokine chronically elevated in obese patients, has been demonstrated to be a cofactor in the progression and severity of AH1N1 influenza leading to acute lung injuries [111] and thought to play a leading role in the development of insulin resistance, hypertension, and cardiovascular diseases [112]. Furthermore, the impaired immune response typical of obese patients can lead to poor vaccination success causing a delay in clearing the viral infection [113]. Given the impact that proinflammatory cytokines can have on glucose metabolism and insulin signaling, it is not surprising that diabetes represents a risk factor in SARS-CoV-2 infection. Furthermore, it may also be possible that a prolonged and acute increase of proinflammatory cytokines could predispose healthy subjects that have been critically affected by COVID-19, to develop diabetes.

## 4. Conclusions

**Figure 1:**
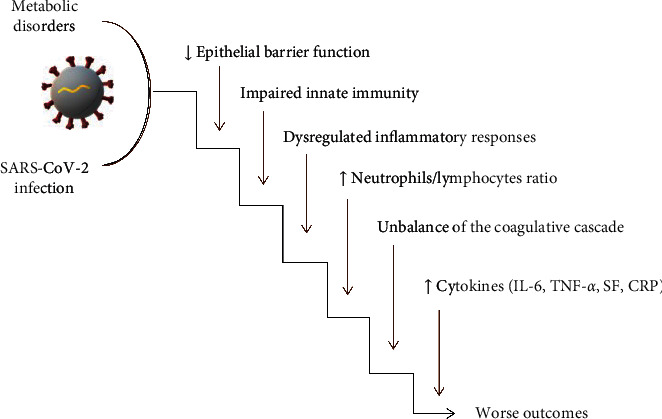
Molecular machineries responsible for the worsening of the SARS-CoV-2 phenotype and prognosis in patients with concomitant metabolic diseases. Biological functions such as inflammation, immunity, and epithelial defense mechanisms are involved.

SARS-CoV-2 patients with concomitant metabolic diseases are at higher risk of worse prognosis and mortality. Molecular mechanisms involving biological functions such as inflammation, immunity, and epithelial defense mechanisms may be implicated in exacerbating the infection leading to worsening clinical outcomes and even death (see Figure 1).

Interestingly, artificial intelligence and data mining approaches have been successfully used to determine the predictive biomarkers for the development of severe infection. An altered ACE2 expression, poor glycemic control, a mild upregulation of liver enzymes, alanine aminotransferase (ALT) and aspartate aminotransferase (AST), high interleukin 6, elevated hemoglobin, and lymphocytopenia are among the clinical presentation that predicts the development of severe COVID-19 infection [14, 114]. This has led to examining the intricate relationship among pathophysiological pathways that may explain why diabetes is a risk factor for COVID-19 and link diabetes with liver disease. It is worth noting that elevated ALT has also been described as a predictor to develop diabetes [115]. Therefore, although clinical reports have shown that diabetic subjects are not at higher risk of SARS-CoV-2 infection compared to healthy subjects, if infected, they are more likely to develop a severe form [116–118]. This knowledge should be extremely useful to guide the clinical management of these patients.

An early diagnosis of metabolic and cardiovascular diseases must be ensured to optimize treatment. The relationship between coexisting metabolic diseases and unfavorable prognosis highlights the importance of rigorous glucose monitoring in COVID-19 patients. In addition, assessment of the coagulation axis and an evaluation of inflammatory markers such as CRP, IL-6, TNF*α*, and serum ferritin, in metabolic patients infected with SARS-CoV-2, should be considered in the decision-making process. Lastly, although studies concerning the impact of antihypertensive and antidiabetic treatments upon COVID-19 course are still ongoing, medication adherence must be promoted to achieve complete control of underlying diseases and to better face the infection. As specific therapies for SARS-CoV-2 are still not available, these considerations have major implications in public health especially for countries affected by a high incidence of metabolic disorders.

## Figures and Tables

**Table 1:** Influence of comorbidities in coronaviruses infections, SARS-CoV, MERS-CoV, and SARS-CoV-2 outbreaks.

	Diabetes (%)	Hypertension (%)	HD (%)	Obesity (%)	References
SARS	2.47–50.0	4.9–19.4	0.9–32.6	—	18–22
MERS	32.4–68.0	28.9–34.0	7.5–28.0	17.0	10, 23
COVID-19	14.3	26.3	16.9	20.7	24, 25

COVID-19 : coronavirus disease 2019; HD: heart disease; MERS: Middle East respiratory syndrome; SARS: severe acute respiratory syndrome.
